# High Prevalence of Uncontrolled Asthma and Its Association With Obesity and GERD‐Related Symptoms in Syria: A Multicenter Cross‐Sectional Study

**DOI:** 10.1002/hsr2.70828

**Published:** 2025-05-21

**Authors:** Duaa Bakdounes, Ruba Dughly, Imad‐Addin Almasri, Nafiza Martini, Majd Hanna, Douaa Albelal, Hussam Al bardan

**Affiliations:** ^1^ Faculty of Medicine Syrian Private University Damascus Syria; ^2^ Stemosis for Scientific Research Damascus Syria; ^3^ Applied Statistics Department Damascus University Damascus Syria; ^4^ Faculty of Medicine Damascus University Damascus Syria; ^5^ Faculty of Medicine Hama University Hama Syria

**Keywords:** asthma control, BMI, gastroesophageal reflux, GERD, obesity/overweight

## Abstract

**Background and Aim:**

Asthma control remains suboptimal globally, with high rates of partly and uncontrolled disease. Poor asthma control can be attributed to the severity of the disease, ineffective treatment, and the presence of comorbid conditions such as obesity and gastroesophageal reflux disease (GERD). This study represents the first investigation in Syria to explore the relationship between asthma control status and common asthma comorbidities, namely obesity and gastroesophageal reflux disease (GERD).

**Methods:**

This multicenter cross‐sectional study interviewed 275 asthma patients from respiratory clinics at four hospitals using questionnaires on sociodemographic, asthma control per Global Initiative for Asthma (GINA) guidelines, symptoms, and management. Body Mass Index (BMI), waist‐to‐hip (WHR) and waist‐to‐height ratios (WHtR) were calculated. Associations were assessed between asthma control, obesity markers, GERD symptoms, and other variables.

**Results:**

Most patients were women (72%) with a mean age of 41 years. Based on GINA criteria, 60% had uncontrolled, 28.7% partly controlled, and only 11.3% well‐controlled asthma. Higher BMI associated significantly with worse control (*p* = 0.006). Unlike WHR, WHtR correlated with poorer asthma control (*p* < 0.001). While GERD diagnosis did not relate significantly to asthma control, symptoms like heartburn, chest pain, and chronic cough did (*p* < 0.05), as did the lack of GERD treatment with Proton Pump Inhibitors (PPI) (*p* = 0.002).

**Conclusion:**

There is a marked prevalence of inadequately controlled asthma in Syria. Both obesity and GERD‐related symptoms correlate with poorer asthma control, emphasizing the need for a comprehensive management strategy.

**Abbreviations**
ACT: asthma control test
BMI: body mass index
COPD: chronic obstructive pulmonary disease
GERD: gastroesophageal reflux disease
GINA: global initiative for asthma
PPI: proton‐pump inhibitor
SABA: short‐acting beta‐agonist
WHR: waist‐to‐hip
WHtR: waist‐to‐height

## 1 Introduction

Asthma, a prevalent and intricate clinical syndrome, affects individuals across all age groups. It is characterized by variable airflow obstruction, bronchial hyperresponsiveness, and airway inflammation, and may manifest in various phenotypes. The symptoms of asthma are nonspecific, complicating its diagnosis in community‐based settings. Regular inhaled corticosteroids (ICS) are the primary treatment of persistent asthma, with inhaled bronchodilators used as rescue medication. Most patients have inadequate asthma control for various reasons, including severe disease, insufficient or ineffective treatment, and the effects of comorbidities [1]. In severe asthma patients, other comorbidities, such as rhino sinusitis and obesity, are a concern, as they could be an underlying cause for asthma symptoms such as wheezing, dyspnea, cough, and chest tightness [2].

Obesity is typically defined quite simply as excess body weight for height. Obesity dramatically increases the risk of chronic disease morbidity—disability, depression, type 2 diabetes, cardiovascular disease, certain cancers—and mortality [3]. Prospective studies indicate that obesity often precedes the onset of asthma. Furthermore, weight gain has been linked to the initiation of asthma symptoms, suggesting a causal relationship between obesity and asthma [4]. Gastroesophageal reflux disease (GERD) is a chronic gastrointestinal disorder that regurgitates gastric contents into the esophagus [5]. The co‐occurrence of gastroesophageal reflux and asthma is quite common, and intricate interactions take place wherein GERD can exacerbate asthma symptoms, or asthma can act as a trigger for, or worsen, GERD [6]. Understanding the relationship between the level of asthma control, obesity, and GERD is crucial for optimizing asthma management strategies. While previous research has explored these associations individually, there remains a need for comprehensive studies that investigate the simultaneous influence of both obesity and GERD on asthma control. Therefore, the aim of this cross‐sectional study is examining the relationship between the level of asthma control, and obesity, and GERD with a particular focus on Syrian patients in Syria, and to provide valuable insights and recommendations for patients suffering from the coexistence of these conditions.

## 2 Materials and Methods

### 2.1 Participants

Over a 5‐month period from October 2022 to February 2023, this multicenter cross‐sectional study enrolled 275 consecutive asthma patients from four distinct hospitals in Syria. To ensure consistency, participants were recruited from specialist respiratory clinics at four hospitals. The study specifically included patients between the ages of 14 and 83 who had been diagnosed with asthma by a hospital or private clinic and according to established clinical guidelines or criteria. Patients with other significant respiratory conditions like chronic obstructive pulmonary disease (COPD) or cystic fibrosis, as well as those who were unable to complete the study interview due to cognitive impairments, were excluded from the study. Before participation, patients were informed about the research objectives and assured that their involvement was voluntary.

After obtaining informed consent, participants were interviewed in thoracic clinic rooms by trained staff using a standardized form developed through a review of medical literature and alignment with Global Initiative for Asthma (GINA) guidelines for assessing asthma control. Assistance was provided during the questionnaire to ensure clarity and understanding, considering the varying education levels among the patients to avoid incorrect data. Height and weight measurements as well as waist‐to‐hip ratio (WHR) and waist‐to‐height ratio (WHtR) were also calculated during visits.

### 2.2 Data Collection

Through interviews, we obtained sociodemographic information about the participants, including age, height, weight, gender, BMI (kg/m^2^), marital status, education level, economic status, occupation, place of residence, and smoking status. Asthma control was assessed in this study based on the GINA symptom control Tool (2022), which is a questionnaire developed by GINA to assess asthma symptom control [7]. It contains four core questions that evaluate the presence of daytime symptoms more than twice a week, nighttime awakenings, activity limitation, and rescue medication use, such as short‐acting beta‐agonists (SABAs) for more than twice a week, over the past 4 weeks. It uses Yes/No responses; if all the questions are answered “no,” the situation is considered “well controlled.” If one or two questions are answered “yes,” then it is considered “partly controlled,” and if three or four questions are answered “yes,” then it is judged “uncontrolled.”

We also collected data on participants' family history of asthma, known triggers, exacerbations, and whether their occupation exacerbated asthma symptoms. The questionnaire also inquired about the medications prescribed, patient adherence to these medications, and the diagnostic methods employed during their initial asthma diagnosis. Regarding GERD, participants were questioned about any previous GERD diagnoses, associated symptoms—including chest pain, chronic cough, hoarseness, and heartburn—and their use of antacids. (Supporting Information S1: The Questionnaire).

### 2.3 Definitions of Variables

Participants' age was determined based on their self‐reported age at the time of the study. Gender was classified as male or female. Marital status was categorized as single, married, or divorced. Economic status of the participants was reported as bad (not enough for basic needs), Acceptable (enough for basic needs), Good (enough for basic needs and some luxuries), or Excellent (enough for basic needs and all luxuries). BMI: A score below 18.5 was considered “underweight”, 18.5–24.9 as “Healthy normal weight”, 25–29.9 as “overweight”, 30 and above as “Obesity”. Education level was classified as uneducated, high school or less, University, or Higher education (PhD and Master's). Occupation status answers' were classified as “working”, “not working”, and “still studying”. As for smoking status, participants were categorized as smokers or non‐smokers. Patients were also asked about medical adherence and the answers were “Yes”, “NO”, or “Occasionally”. Place of residence included “Urban” or “Rural”. For Waist to hip ratio: In men, a score of 0.95 or lower was considered as “Low risk”, 0.96–1.00 was considered “Moderate risk”, and 1.0 or higher as “High risk”. In women, a score of 0.80 or lower was considered as “Low risk”, 0.81–0.85 as “Moderate Risk”, and 0.86 or higher as “High Risk”. For waist to height ratio: In both men and women, scores of 0.43–0.46 were considered “underweight”, 0.46–0.53 as “Normal weight”, 0.53–0.58 as “Overweight”, and 0.58–0.63 as “Obesity”.

### 2.4 Sample Size

The sample size (*n*) was determined by Cochran's sample size formula with the assumption of 95.5% confidence level (*Z* = 2), *e* is the margin of error which is 10%, *p* is the (estimated) proportion of the population which has the attribute in question, and it equals 50% (or 0.5), and *q* is 1−*p*.

$$n=\frac{z^{2}\mathrm{pq}}{{\mathrm{e}}^{2}}=\frac{{\left(2\right)}^{2}*0.5*0.5}{{\left(0.1\right)}^{2}}=100$$

The required sample size (*n*) for this study, applying the previous formula, is at least 100.

If we adopt a 5% margin of error, the sample size becomes 400.

$$n=\frac{z^{2}\mathrm{pq}}{{\mathrm{e}}^{2}}=\frac{{\left(2\right)}^{2}*0.5*0.5}{{\left(0.05\right)}^{2}}=400$$

Therefore, with a confidence of 95.5 and an error ranging from 5% to 10%, a sample size ranging from 100 to 400 individuals should be required. We complied with these requirements and collected 275 participants [8].

### 2.5 Data Analysis

The information gathered from the paper questionnaires was input into SPSS version 25 for analysis. Descriptive analysis, which involved calculating frequencies and percentages, was carried out for all variables in the study. The mean, median, mod, and standard deviation were used to describe quantitative variables, as well as the maximum and minimum values. In our study, the variables BMI and WHR did not follow a normal distribution (*p* < 0.05). While WHtr demonstrated normality (*p* = 0.081). Based on these findings, we employed the *χ* ^2^ test to analyze associations between categorical variables, and the Spearman's rank correlation coefficient test to examine relationships between ranked or ordinal variables. In all cases, statistical significance was defined as a *p* value of less than 0.05.

## 3 Results

### 3.1 Study Population

This cross‐sectional study included 275 asthma patients. The majority were women (72%) and non‐smokers (64.4%). Most were married (58.2%), and some had a university education (29.5%). The mean age was 41 years. In terms of economic status, nearly half of the participants (43.3%) lacked the resources to fulfill their basic needs. The average body mass index (BMI) was 28.2 kg/m^2^. Based on BMI, 2.5% were underweight, (32.4%) were average weight, 33.5% were overweight, and 31.6% were obese. Regarding the waist‐to‐hip ratio results, among the 170 participants, 108 individuals (63.5%) were found to have a high health risk, 16 individuals (9.4%) had a moderate risk, and 46 individuals (27.1%) had a low risk. In addition, based on the Waist‐to‐Height Ratio (WHtR), out of 259 individuals, 174 were classified as being overweight or obese, 69 were categorized as normal weight, and 16 were identified as underweight. Most patients (83.6%) reported that their work did not affect their asthma. Approximately half of the patients (51.3%) reported an absence of asthma in their family history. In addition, most patients (81.8%) had allergies to some substances, and 8.36% did not receive any asthma treatment. In terms of medication adherence, 40% reported high adherence, while 32% had only occasional adherence (Table 1).

**Table 1:** Sociodemographic and other characteristics of participants, asthma control, and GERD assessment.

Sociodemographic info.		
275 (100%)	Mean (40.96)	Age
198 (72%)	Female	Gender
77 (28%)	Male	
105 (38.2)	Rural	Place of residence
170 (61.8%)	Urban	
95 (34.5%)	Single	Marital status
160 (58.2%)	Married	
20 (7.3%)	Divorced	
119 (43.3%)	Bad	Economic status
64 (23.3%)	Acceptable	
65 (23.6%)	Good	
27 (9.8%)	Excellent	
43 (15.6%)	Uneducated	Education level
138 (50.2%)	High school and less	
81 (29.5%)	University	
13 (4.7%)	Higher education	
115 (41.8%)	Working	Occupation status
103 (37.5%)	Not working	
57 (20.7%)	Still studying	
7 (2.5%)	Underweight	BMI
89 (32.4%)	Normal weight	
92 (33.5%)	Overweight	
87 (31.6%)	Obese	
98 (35.6%)	Smoker	Smoking status
177 (64.4%)	Nonsmoker	
110 (40%)	Yes	Medication adherence
88 (32%)	Occasionally	
77 (28%)	No	
225 (81.8%)	Yes	Having allergies
14 (5.1%)	Maybe	
36 (13.1%)	No	
45 (16.4%)	Yes	Work affects asthma
230 (83.6%)	No	
134 (48.7%)	Yes	Family history of asthma
141 (51.3%)	No	
252 (91.64%)	Yes	Taking medication
23 (8.36%)	No	
108 (63.5%)	High health risk	Waist‐to‐hip ratio^a^
16 (9.4%)	Moderate risk	
46 (27.1%)	Low risk	
16 (6.2%)	Underweight	Waist‐to‐Height Ratio (WHtR)^b^
69 (26.6%)	Normal weight	
101 (39%)	Overweight	
73 (28.2%)	Obese	
Asthma control		
165 (60%)	Uncontrolled	Asthma control based on GINA assessment
79 (28.7%)	Partly controlled	
31 (11.3%)	Well controlled	
74.5%	Yes	In the past 4 weeks, have your asthma symptoms more than twice/week?
25.5%	No	
172 (62.5%)	Yes	In the past 4 weeks, have your asthma symptoms awakened you at night or caused you to wake up earlier than usual in the morning?
103 (37.5%)	No	
201 (73.1%)	Yes	During the past 4 weeks, has your asthma limited your ability to participate in activities? For example, avoiding rooms with smoke, perfume, or cooking odors, or feeling fatigued while walking or exercising?
74 (26.9%)	No	
134 (48.7%)	Yes	In the past 4 weeks, have you needed to use a nebulizer, nasal spray, or a blue inhaler?
141 (51.3%)	No	
GERD Assessment		
89 (32.4%)	Chest pain	GERD symptoms
84 (30.5%)	Hoarseness	
88 (32%)	Chronic cough	
47 (17.1%)	Pharyngeal inflammation	
23 (8.4%)	Laryngeal inflammation	
118 (42.9%)	Heartburn	
114 (41.5%)	Stomach pain	
85 (30.9%)	Acidity in the mouth	
7 (2.5%)	Lung injuries	
119 (43.3%)	Yes	Taking medication for GERD
156 (56.7%)	No	
30 (10.9%)	Yes	Prior diagnosis of GERD
245 (89.1%)	No	

^a^ *n* = 170 participants.

^b^ *n* = 259 participants.

### 3.2 Asthma Control

Asthma control was assessed using GINA assessment. Over the past 4 weeks, 60% had uncontrolled asthma, 28.7% partly controlled, and 11.3% well controlled. The majority experienced frequent asthma symptoms ( > 2 times/week; 74.5%), nighttime awakenings (62.5%), and activity limitation (73.1%), and half required frequent rescue inhaler use (48.7%) (Table 1).

### 3.3 GERD Assessment

Most patients (89.1%) were not previously diagnosed with GERD. Yet, common GERD symptoms were reported, including heartburn (42.9%), stomach pain (41.5%), chest pain (32.4%), and chronic cough (32%). Over half of the participants (56.7%) did not take medication for reflux (Table 1).

### 3.4 Principle Associations

Our study findings showed that there was no significant association between asthma control and smoking status (*p* = 0.143) or occupation (*p* = 0.744). In addition, higher body mass index (BMI) was associated with worse asthma control. As BMI increased, asthma control worsened, as evidenced by a statistically significant inverse correlation (correlation coefficient = −0.164, *p* = 0.006). The study also found a significant inverse association between asthma control and waist‐to‐height ratio (WHtR) (*p* < 0.001, Correlation Coefficient = −0.255). As WHtR increased, asthma control worsened. However, no significant correlation was observed between asthma control and waist‐to‐hip ratio (WHR) (*p* = 0.264). Furthermore, frequent asthma symptoms, night symptoms, activity limitation, and rescue use were also significantly associated with worse asthma control (all *p* < 0.001). While a formal diagnosis of GERD did not show a statistically significant association with asthma control (*p* = 0.168), specific symptoms commonly associated with GERD did demonstrate significant relationships with worse asthma control. These symptoms included heartburn, chest pain, and chronic cough (all *p* < 0.05). Moreover, lack of reflux treatment with Proton‐pump inhibitors (PPIs) was associated with worse control (*p* = 0.002) (Tables 2, 3, 4). GERD were also found to be related with both WHtR (*p* value = 0.033) and WHR (*p* value = 0.031).

**Table 2:** The relation between asthma control and some variables (*χ* ^2^ test).

*p*‐value	Variables
0.744	Relation between occupation type and poor asthma control
0.000	Relation between asthma control and frequent symptoms for more than twice a week in the past 4 weeks
0.000	Relation between asthma control and night symptoms in the past 4 weeks
0.000	Relation between asthma control and activity limitation in the past 4 weeks
0.000	Relation between asthma control and the need for rescue use symptoms in the past 4 weeks
0.168	Relation between asthma control and prior diagnoses of GERD
0.001	Relationship between asthma control and GERD symptoms (chest pain, hoarseness, chronic cough, laryngeal inflammation, heartburn, acidity in the mouth, stomach pain, lung injury, pharyngeal inflammation, respectively)
0.000	
0.000	
0.005	
0.050	
0.005	
0.028	
0.617	
0.253	
0.002	Relation between asthma control and GERD untreated with PPI.

**Table 3:** The relationship between variables (*χ* ^2^ test).

*p* value	*χ* ^2^ test	Variables
0.512	5.252	Relationship between GERD and BMI
0.033	13.743	Relationship between GERD and WHtR
0.271	2.165	The relationship between medication adherence and montelukast treatment
0.829	0.376	Relationship between medication adherence and bronchodilator therapy
0.002	12.540	Relationship between medication adherence and systemic spray therapy
0.800	1.650	Studying the relationship between the degree of asthma control and GERD treated with PPI drugs
0.006	14.539	Studying the relationship between the degree of asthma control and GERD not treated with PPI medications

**Table 4:** The relation between asthma control and some variables (Spearman's rho test).

Sig. (2‐tailed) < 0.05	Correlation coefficient	Variables
0.006	−0.164	BMI
0.000	−0.255	WHtR
0.004	−0.173	Specific body mass index
0.031	−0.165	Relationship between GERD and WHR
0.143	0.089	Smoking
0.264	0.086	WHR

Based on Table 5, the following observations can be made:

**Table 5:** Multivariable logistic regression.

	Estimate	Std. error	Wald	df	Sig	95% Confidence interval	
						Lower bound	Upper bound
BMI	−0.039	1.944	0.000	1	0.984	−3.848	3.771
WHTR	0.041	1.892	0.000	1	0.983	−3.667	3.748
In the past 4 weeks, have your asthma symptoms more than twice/week?	11.377	4.005	8.068	1	0.005	3.527	19.227
In the past 4 weeks, have your asthma symptoms awakened you at night or caused you to wake up earlier than usual in the morning?	11.219	3.941	8.103	1	0.004	3.494	18.943
During the past 4 weeks, has your asthma limited your ability to participate in activities? For example, avoiding rooms with smoke, perfume, or cooking odors, or feeling fatigued while walking or exercising?	11.793	4.060	8.438	1	0.004	3.836	19.750
In the past 4 weeks, have you needed to use a nebulizer, nasal spray, or a blue inhaler?	10.838	4.143	6.843	1	0.009	2.718	18.959
Chest pain	−0.158	3.501	0.002	1	0.964	−7.020	6.704
Hoarseness	0.370	4.327	0.007	1	0.932	−8.111	8.850
Chronic cough	0.217	3.733	0.003	1	0.954	−7.098	7.533
Pharyngeal inflammation	−0.076	7.230	0.000	1	0.992	−14.247	14.094
Heartburn	−0.012	4.589	0.000	1	0.998	−9.007	8.982
Acidity in the mouth	−0.124	4.259	0.001	1	0.977	−8.472	8.224
Stomach pain	0.406	3.620	0.013	1	0.911	−6.688	7.500
GERD	0.273	3.516	0.006	1	0.938	−6.618	7.164

1. For categorical BMI, the *p* value (0.984) exceeds 0.05, showing no statistically significant association. Hence, categorical BMI does not affect asthma control.

2. The *p* value for categorical WHTR (0.983) is greater than 0.05, indicating no significant link with asthma control.

5. The *p* value for the question, “In the past 4 weeks, did you experience asthma symptoms more than twice a week?” (0.005), is less than 0.05, indicating a statistically significant effect on asthma control.

6. The *p* value for the question, “In the past 4 weeks, did asthma symptoms wake you up at night or earlier in the morning?” (0.004), is below 0.05, signifying a significant relationship with asthma control.

7. The question, “In the past 4 weeks, did asthma prevent you from certain activities or cause fatigue during exercise?” has a *p*‐value of 0.004, indicating a significant effect.

8. The *p*‐value for the question, “In the past 4 weeks, did you need a nebulizer or rescue inhaler?” (0.009), is less than 0.05, showing a significant effect on asthma control.

9. The symptom “chest pain” has a *p*‐value of 0.964, which is above 0.05, indicating no significant effect on asthma control.

10. The *p* value for “hoarseness” (0.932) suggests no statistically significant relationship with asthma control.

11. The *p* value for “chronic cough” (0.954) exceeds 0.05, showing no significant effect.

12. “Laryngitis” has a *p* value of 0.992, indicating no significant impact on asthma control.

13. The *p* value for “heartburn” (0.998) suggests no relationship with asthma control.

14. The symptom “acid taste in the mouth” has a *p* value of 0.977, showing no significant relationship.

15. The *p* value for “epigastric pain” (0.911) is above 0.05, indicating no significant effect on asthma control.

16. The variable “GERD (Gastroesophageal Reflux Disease)” has a *p* value of 0.938, indicating no significant effect on asthma control.

## 4 Discussion

Asthma imposes a substantial global disease burden, affecting hundreds of millions of people worldwide [9]. To our knowledge, this is the first study to characterize asthma control and associated factors in Syrian population. Research conducted across diverse global regions including North America, Europe, Asia, and the Middle East, has consistently demonstrated that asthma control remains suboptimal for many patients [10, 11, 12, 13, 14, 15, 16, 17, 18, 19, 20, 21]. High rates of uncontrolled disease have been reported in Nigeria (82.9%), the Maghreb region (71.3%), and Ethiopia (71.4%) [22, 23, 24].

The current study's findings align with these prior studies, with 60% of patients exhibiting uncontrolled asthma. The high rate of uncontrolled asthma observed in this specialty clinic may not fully reflect the true extent of inadequate asthma management in primary care, especially in countries like Syria. Factors related to the difficult economic situation, such as a shortage of hospitals, particularly in rural areas, mean people often seek primary care not just for routine checkups but also for more severe health issues. Since 61.8% of our sample came from urban areas while only one‐third were from rural areas, the true scale of poorly controlled asthma at the primary care level may be underrepresented. Additionally, a lack of asthma specialists may also play a significant role in the high rate of uncontrolled asthma. Previous research has shown that asthma patients treated by specialists tend to have better outcomes than those solely seen in primary care [25, 26]. Therefore, if this clinical population already exhibits a high burden of uncontrolled disease, the problem may be even more pervasive among primary care asthma patients in the region who lack access to dedicated respiratory care.

This study's finding that most asthma patients were women (72%) is consistent with previous studies, especially those focusing on the postpuberty period [27, 28, 29]. Population research by Leynaert et al. demonstrated a 20% higher asthma prevalence in women versus men over the age of 35 [30]. Longitudinal studies by Mandhane et al. also found that girls consistently showed greater asthma prevalence than boys from early childhood through adolescence. These findings suggest that female sex is a risk factor across the lifespan [31]. The mechanism may relate to female hormonal influences—multiple studies indicate that estrogens and progesterone can impact asthma [32]. The skew towards women asthma patients in the current Syrian cohort mirrors this female predominance seen previously in other nations. This highlights the global nature of sex‐based differences in asthma frequency, with the female sex conferring a disproportionate asthma burden.

Numerous studies have linked higher body mass index (BMI) to worse asthma control, and the positive association between BMI and uncontrolled asthma observed here reinforces these conclusions [25, 29, 33, 34, 35, 36]. Moreover, our study revealed a strong association between elevated abdominal adiposity, as indicated by WHtR, and suboptimal asthma control (*p* < 0.001), which is consistent with findings from other studies [37, 38]. The presence of excess abdominal fat can adversely affect various respiratory parameters, including chest wall compliance, respiratory muscle strength and function, lung volumes, and peripheral airway diameter. Consequently, these physiological changes may contribute to increased airway hyperresponsiveness and exacerbation of asthma symptoms [38]. According to research by Boulet and Franssen, obese asthma patients were less likely to attain adequate asthma control compared to nonobese patients when treated with inhaled corticosteroids alone or in combination with long‐acting beta‐agonists indicating that obesity may impair response to standard asthma controller medications [39]. The underlying mechanisms for decreased asthma medication efficacy in obese patients may involve higher levels of inflammatory cytokines that can inhibit the activity of mitogen‐activated protein kinase phosphatase‐1, a key signaling protein influenced by glucocorticoids, which in return could impair patient responses to inhaled and oral steroid treatments [40]. Besides, research suggests that abdominal visceral adipose tissue exhibits a higher pro‐inflammatory state compared to abdominal and gluteal subcutaneous adipose tissue [38]. In our study, unlike WHtR, WHR was not found to be correlated with poorer asthma control. There are a few potential explanations for this finding. WHtR is more sensitive to central obesity and can distinguish between subcutaneous and visceral fat compared to WHR. Visceral fat, located around the organs, is more metabolically active and can have a greater impact on respiratory function and asthma control. In addition, abdominal fat, which is measured better by WHtR, can directly affect lung function by reducing lung volume, decreasing diaphragm movement, and increasing chest wall resistance. These factors are less influenced by hip circumference, which is part of the WHR calculation. However, the existing studies examining the relationship between measurements of obesity (such as BMI, WHtR, WHR) and asthma control have yielded conflicting results, highlighting the necessity for further research to better understand this intricate association [41, 42].

In addition, some comorbidities of obesity such as GERD may also have an effect on asthma, our study found a strong correlation between GERD with both WHR and WHtR. Prior studies suggest gastroesophageal reflux can exacerbate asthma by increasing cough sensitivity and that proper GERD management often improves asthma control [35, 43, 44, 45]. Furthermore, asthma can be triggered by reflux either directly, through its impact on the airway via an aspiration‐induced response, or indirectly, through the activation of neurogenic inflammation [46]. However, in our study, there was no significant link between GERD diagnosis and level of asthma control, despite strong associations between several reflux‐related symptoms and worse control. This discrepancy suggests potential underdiagnoses of GERD in this study, as patients may have failed to report the condition while acknowledging related signs. Undiagnosed reflux could, therefore, still negatively impact their asthma. Additionally, the link found between a lack of GERD treatment with PPIs and worse asthma control reinforces that an underlying connection likely exists. This highlights the need for appropriate reflux therapy to potentially improve asthma outcomes, even if patients do not report overt GERD, as prior studies reported that treating reflux can successfully improve asthma control, especially in those with severe reflux symptoms [47]. However, other studies found no association between asthma control status and GERD [48, 49, 50].

Unlike previous studies demonstrating associations between smoking and worse asthma control and outcomes [51, 52, 53], this study did not find a significant relationship between smoking status and asthma control, with a *p* value of 0.143. Similarly, while prior research has identified occupational exposures as risk factors for poor control [54, 55], this study failed to find any association with occupation. The lack of broad occupational classifications and more details about smoking in this study questionnaire may have contributed to the discordant findings. Despite the lack of significant associations in this study, smoking, and occupational exposures likely represent undetermined risk factors that require further investigation with more robust characterization to fully elucidate their relationships with asthma control.

In terms of socioeconomic status, this study found that 43.3% of participants experienced poor economic status without adequate resources for basic needs. Only 34.2% attained a university or higher education level. Prior research by Bacon et al. has linked lower socioeconomic status, assessed by education level, to worse asthma control and overall morbidity [56]. Additionally, an observational study of over 4000 low‐income asthma patients associated having health insurance with improved asthma outcomes [57]. The socioeconomic disadvantages faced by a substantial portion of the current Syrian study sample—especially during the country's crisis—may contribute to the high levels of inadequate asthma control observed.

### 4.1 Strengths and Limitations

This study marks the first investigation of its kind conducted within the Syrian population. Moreover, it explores the relationship between asthma control and obesity by employing three distinct measurements: Body Mass Index (BMI), Waist‐to‐Hip Ratio (WHR), and Waist‐to‐Height Ratio (WHtR), by considering multiple measurements, the study provides a more nuanced understanding of the relationship. However, it has some restrictions that should be noted. Dependence on participant self‐reporting for data could allow errors in recall and reporting to skew the findings. There is also the possibility of sampling bias since patients with more severe illnesses may have been more inclined to take part in the clinics. Another limitation arises from reliance on GERD‐related symptoms instead of a precise diagnosis of GERD, primarily due to the poor economic situation in Syria, which hinders the use of Upper gastrointestinal (GI) endoscopy and esophageal pH testing. This can affect the establishment of a relationship between asthma control and GERD.

Our study did not account for treatment doses, as there was significant variability in dosages among the patients in our sample, along with differences in treatment methods.

The inquiry focused broadly on treatment adherence rather than specific details about the treatments themselves. Unfortunately, we were unable to delve deeper into treatment specifics due to patients' limited awareness and the economic and social challenges that often hinder their ability to purchase medications. Additionally, we were unable to address potential confounding factors such as treatment variations and the use of inhaled corticosteroids (ICS).

## 5 Conclusion

Our cross‐sectional study sheds light on the intricate relationships between asthma, obesity, and gastroesophageal reflux disease (GERD) among Syrian patients, offering valuable insights into the factors contributing to the high prevalence of uncontrolled asthma in the region. The findings underscore the need for a comprehensive understanding of the interplay between these comorbidities to optimize asthma management strategies. Notably, the study identifies a significant proportion of patients with uncontrolled asthma, particularly those attending specialty clinics, suggesting a potentially more widespread issue at the primary care level and underscoring the importance of targeted interventions. The study also reveals a disproportionately higher prevalence of asthma among women and individuals facing socioeconomic disadvantages, underscoring the multifaceted nature of the asthma burden in Syria. While smoking and occupational exposures did not show significant associations with asthma control in this study, they warrant further investigation with more robust characterization. The positive correlation between body mass index (BMI) and uncontrolled asthma aligns with existing literature, emphasizing the impact of obesity on asthma outcomes. The waist‐to‐height ratio emerged as a more relevant anthropometric measure for assessing the impact of central obesity on asthma control compared to the waist‐to‐hip ratio. Further research is needed to fully understand the complex relationship between body composition, fat distribution, and asthma outcomes. Contrarily, the lack of a significant link between GERD diagnosis and asthma control, despite associations with reflux‐related symptoms, suggests potential underdiagnosis and emphasizes the need for heightened awareness and appropriate reflux therapy.

## Author Contributions

**Duaa Bakdounes:** conceptualization, writing – original draft, writing – review and editing, methodology, and resources. **Ruba Dughly:** conceptualization, writing – original draft, writing – review and editing, methodology, and resources. **Imad‐Addin Almasri:** conceptualization, formal analysis, data curation, writing – review and editing, and methodology. **Nafiza Martini:** conceptualization, writing – original draft, methodology, writing – review and editing, project administration, and resources. **Majd Hanna:** conceptualization, writing – original draft, methodology, writing – review and editing, and resources. **Douaa Albelal:** writing – original draft, writing – review and editing, and resources. **Hussam Al bardan:** supervision, conceptualization, writing – review and editing, and methodology.

## Ethics Statement

The Ethical Committee approved this study in the Faculty of Medicine at Sham Private University, Syria, with the number (6130). On October 16, 2022, all participants gave their written informed consent to participate voluntarily before their inclusion in the study. The study design and conduct aligned with the ethical principles of the Declaration of Helsinki [58].

## Consent

Written informed consent was obtained from the patients for publication and any accompanying images. A copy of the written consent form is available for review by the editor‐in‐chief of this journal upon request.

## Conflicts of Interest

The authors declare no conflicts of interest.

## Transparency Statement

The lead author Nafiza Martini affirms that this manuscript is an honest, accurate, and transparent account of the study being reported; that no important aspects of the study have been omitted; and that any discrepancies from the study as planned (and, if relevant, registered) have been explained.

## Supporting information

The Questionnaire.

## Data Availability

The datasets collected and analyzed during the current study are not publicly available. Some restrictions apply to the availability of these data, but they are available from the corresponding author on reasonable request.
