# Supplementary material for: High Prevalence of Uncontrolled Asthma and Its Association With Obesity and GERD‐Related Symptoms in Syria: A Multicenter Cross‐Sectional Study
Source: Health Sci Rep. 2025 May 21;8(5):e70828. doi: 10.1002/hsr2.70828 (PMC12095846; doi:10.1002/hsr2.70828)
Supplement: Supplementary file 1 — The Questionnaire. [file HSR2-8-e70828-s001.docx]

The purpose of this study is to characterize the level of asthma control among individuals in our region and examine how it may relate to obesity and acid reflux disease. We will be interviewing you in order to collect details about your demographics, medical history, asthma symptoms and attacks, triggers, treatments being used, and other health conditions you may have. We will also be taking some physical measurements like height, weight, waist circumference.

Your participation is completely voluntary. You can choose whether to take part and whether to answer any given question. There will be no impacts to your care if you decide not to participate or choose to withdraw. You may skip questions you do not want to answer.

We greatly appreciate your willingness to take part in this important health research study. The success of this endeavor relies on the generous involvement of patients like you. Together we can expand knowledge of optimal asthma care.

| I agree to participate in the research | ⭘ Yes | ⭘ NO |
| --- | --- | --- |

| Socio-demographic Info. | | | | | |
| --- | --- | --- | --- | --- | --- |
| Gender | ⭘ man | ⭘ women | Hip Circumference  Waist Circumference | |  |
| Age (in years) | ………………………………. |  | Height (in cm)  Weight (in Kg) | | ……………………………….  ………………………………… |
| marital status | ⭘ Single | ⭘ Married | - Divorced |  |  |
| The Hospital in which the patient was | ⭘ Ibn-Alnafees | ⭘ Al-Moujtahed | - Al-Mowasat | - Red Crescent |  |
| Economic situation | ⭘Weak  (Not enough for basic needs) | ⭘Intermediate  (for basic needs only) | ⭘Good (for basic needs and some luxuries) | ⭘Excellent  (for basic needs and luxuries) |  |
| Education level | ⭘ uneducated | ⭘ Primary | ⭘ Secondary | ⭘ Undergraduate | - Master/PhD |
| Work Status | ⭘ employed | ⭘ Self-employed | ⭘ Do not work, still student | | Do not work |
| Place of Residence | ⭘ Rural | ⭘ Urban |  | | |
| Smoking Habits | - Smoker | - Non-Smoker |  | | |

| Asthma-Related Questions | | | | | | | | | | | | | | | |
| --- | --- | --- | --- | --- | --- | --- | --- | --- | --- | --- | --- | --- | --- | --- | --- |
| Does your occupation involve working with substances or environments that may aggravate asthma symptoms? | | | ⭘Yes | | ⭘No | | |  | | | | |  |  | |
| Is there anyone in your family who has been diagnosed with asthma? | | | ⭘Yes | | ⭘No | | |  | | | | |  |  | |
| Do you have a sensitivity to any of the following smells: smoke, cooking odors, dust, chlorine, detergents, or strong perfumes? | | | ⭘Yes | | ⭘No | | |  | | | | |  |  | |
| Have you received a formal diagnosis of asthma from a chest specialist? | | | ⭘Yes | | ⭘No | | |  | | | | |  |  | |
| What criteria did the doctor use to diagnose your asthma? | | | ⭘Symptoms (Cough, dyspnea..) | | ⭘Skin Prick Test | | | - Lung Function tests (Spirometry) | | | | | ⭘Pulmonary stress Test | Stethoscope and clinical examination | |
| What treatment options did the doctor recommend for managing your asthma? | | | ⭘Montelukast | | ⭘Bronchiodilators | | | - Nasal corticosteroids | | | | |  |  | |
| Are you currently adhering to the prescribed medication for your asthma? | | | ⭘Yes | | ⭘No | | | - Sometimes | | | | |  |  | |
| How would you rate the control of your asthma symptoms over the past four weeks? | | | ⭘Uncontrolled | | ⭘Poor controlled | | | - Partly controlled | | | | | ⭘ Well controlled | - Completely controlled | |
| GINA Questionnaire | | | | | | | | | | | | | | | |
| Over the past four weeks, have you experienced asthma symptoms such as wheezing, coughing, shortness of breath, chest tightness, or chest pain more than twice a week? | | | - Yes | | | | | | | - No | | | | | |
| In the past four weeks, have your asthma symptoms awakened you at night or caused you to wake up earlier than usual in the morning? | | | - Yes | | | | | | | - No | | | | | |
| During the past four weeks, has your asthma limited your ability to participate in activities? For example, avoiding rooms with smoke, perfume, or cooking odors, or feeling fatigued while walking or exercising? | | | - Yes | | | | | | | - No | | | | | |
| In the past four weeks, have you needed to use a nebulizer, nasal spray, or a blue inhaler? | | | - Yes | | | | | | | - No | | | | | |
| Gastroesophageal Reflux Disease (GERD) Questions | | | | | | | | | | | | | | | |
| Have you received a diagnosis of gastroesophageal reflux disease (GERD), which involves stomach acid flowing back into the esophagus? | - **Yes** | | | | | | | | - No | | | | | | |
| Which of the following symptoms associated with GERD, do you experience? | Chest Pain | Chronic Cough | | Hoarseness | | heartburn | Laryngitis | | Pharyngitis | | Stomach ache | Bitter/sour taste | | | Lung Damage |
| Are you currently taking any stomach-protecting medications, such as Azytom, Liberex, Omebral, Maalox, Pantoprazole, Rabeprazole, Lansoprazole, Risic, or others? | - Yes | | | | | | | | - NO | | | | | | |
